# Fc-mediated activity of EGFR x c-Met bispecific antibody JNJ-61186372 enhanced killing of lung cancer cells

**DOI:** 10.1080/19420862.2016.1249079

**Published:** 2016-10-27

**Authors:** Katharine D. Grugan, Keri Dorn, Stephen W. Jarantow, Barbara S. Bushey, Jose R. Pardinas, Sylvie Laquerre, Sheri L. Moores, Mark L. Chiu

**Affiliations:** a Biologics Research, Janssen Research and Development, LLC, Spring House, PA, USA; b Oncology, Janssen Research and Development, LLC, Spring House, PA, USA

**Keywords:** Antibody-dependent cell mediated cytotoxicity, antibody-dependent cell mediated phagocytosis, bispecific, c-Met, EGFR, Fc engineering, hemi-afucosylated, low fucose

## Abstract

Epidermal growth factor receptor (EGFR) mutant non-small cell lung cancers acquire resistance to EGFR tyrosine kinase inhibitors through multiple mechanisms including c-Met receptor pathway activation. We generated a bispecific antibody targeting EGFR and c-Met (JNJ-61186372) demonstrating anti-tumor activity in wild-type and mutant EGFR settings with c-Met pathway activation. JNJ-61186372 was engineered with low fucosylation (<10 %), resulting in enhanced antibody-dependent cell-mediated cytotoxicity and FcγRIIIa binding. In vitro and in vivo studies with the single-arm EGFR or c-Met versions of JNJ-61186372 identified that the Fc-activity of JNJ-61186372 is mediated by binding of the anti-EGFR arm and required for inhibition of EGFR-driven tumor cells. In a tumor model driven by both EGFR and c-Met, treatment with Fc-silent JNJ-61186372 or with c-Met single-arm antibody reduced tumor growth inhibition compared to treatment with JNJ-61186372, suggesting that the Fc function of JNJ-61186372 is essential for maximal tumor inhibition. Moreover in this same model, downregulation of both EGFR and c-Met receptors was observed upon treatment with Fc-competent JNJ-61186372, suggesting that the Fc interactions are necessary for down-modulation of the receptors in vivo and for efficacy. These Fc-mediated activities, in combination with inhibition of both the EGFR and c-Met signaling pathways, highlight the multiple mechanisms by which JNJ-61186372 combats therapeutic resistance in EGFR mutant patients.

## Abbreviations

ADCC: antibody-dependent cell mediated cytotoxicity
ADCP: antibody-dependent cell mediated phagocytosis
PBMC: peripheral blood mononuclear cell
bsAB(s): bispecific antibody(s)
cFAE: controlled Fab-arm exchange
EGFR: epidermal growth factor receptor
c-Met: hepatocyte growth factor receptor

## Introduction

Lung cancer causes more deaths than any other type of cancer, and XYZ to the American Cancer Society estimated that over 200,000 new cases of lung cancer were diagnosed in 2015.^1^ Non-small cell lung cancer (NSCLC) accounts for 83% of all lung cancer cases with epidermal growth factor receptor (EGFR)-activating mutations frequently found in these tumors (10–15% in Caucasians, 50% in Asians).^2^ EGFR is a transmembrane receptor tyrosine kinase that, upon activation by ligand or mutation, initiates signaling cascades that enhance cell proliferation, invasion, survival and angiogenesis.^3^ EGFR mutations result in enhanced sensitivity to EGFR tyrosine kinase inhibitors (TKI), which are an approved first-line treatment for stage IV NSCLC.^4^ Despite good initial response rates (70–80%), resistance to TKI therapies within 1 y of treatment is common and has been attributed to secondary EGFR mutations (EGFR^T790M^), as well as activation of c-Met signaling through MET gene amplification, overexpression of c-Met protein, or an increased local concentration of its ligand HGF.^5-8^

C-Met signaling mediates invasive growth^9^ activated upon binding of the paracrine factor hepatocyte growth factor (HGF) to the receptor tyrosine kinase c-Met expressed on epithelial cells. This pattern of cell migration, invasion, and survival, which is critical during development and wound healing, is often dysregulated in tumorigenesis.^9^ EGFR and c-Met are frequently co-expressed in tumors and the 2 pathways converge on similar downstream signaling mediators such as ERK/MAPK and PI3K/AKT. Cross-talk between EGFR and c-Met in lung cancer, including EGFR-dependent phosphorylation and activation of c-Met through EGFR ligands, has been widely reported.^10-12^ Because of this cross-talk and link between EGFR TKI resistance and c-Met activation, simultaneous targeting of these 2 pathways holds promise.^13,14^ Multiple studies have highlighted the use of EGFR and c-Met combination treatments, as well as single, dual targeting therapeutics.^3,15-17^ In response to this need, we designed JNJ-61186372 as a bispecific antibody to dually target EGFR and c-Met.^18^

Monoclonal antibodies (mAb) mediate tumor cell inhibition through multiple mechanisms of action, including direct target inhibition through Fab arm engagement, induction of apoptosis and Fc-mediated cell depletion by immune cells.^19^ Once a mAb binds to a tumor cell, the Fc portion of the mAb can recruit and activate components of the complement system and innate immune effector cells, including monocytes, macrophages, natural killer (NK) cells, and neutrophils, resulting in lysis and destruction of the mAb-targeted cancer cell through complement-dependent cytotoxicity (CDC), antibody-dependent cell-mediated cytotoxicity (ADCC) or antibody-dependent cellular phagocytosis (ADCP).^19-22^ Evidence from multiple in vivo models suggests that antibody binding to cellular Fc gamma receptors (FcγRs) is the major pathway for antibody-dependent cytotoxicity.^23,24^

The potency of Fc-mediated immune effector mechanisms by therapeutic antibodies is dependent on the binding affinities to both the target ligand(s) and the activation of FcγRs.^25^ Engineering within the Fc has identified critical residues important for FcγR binding. An Fc variant of IgG2, designated as IgG2σ, was engineered with multiple amino acid substitutions within the hinge/CH2 region of the Fc, resulting in reduced affinity for Fcγ receptors and C1q complement protein and lack of immune effector activity.^26^ Alterations of the carbohydrate profile of the Fc can also affect Fc-mediated function. Within the Fc, an N-linked glycan attaches at Asn297 and consists of a core structure containing n-acetyl-D-glucosamine (GlcNAc) and mannose. Additional heterogeneous modifications, including the addition of fucose, bisecting GlcNAc, galactose, and sialic acid, can result in more than 30 variant forms.^25^ Batch-to-batch variations in glycan profile are common during the manufacturing process of therapeutic mAbs. The N-glycan stabilizes particular conformations of the CH2 domain, allowing for increased accessibility and tighter binding to the FcγRs. Deglycosylated antibodies retain antigen and protein A binding similar to wild-type antibody, but are defective in binding to FcγRs, stabilizing complement, and induction of ADCC.^25^ Antibodies produced in cell lines engineered to generate antibodies with afucosyl N-linked glycans have enhanced binding to FcγRIIIa and increased in vitro ADCC activity.^8,27^ It has been suggested that fucose-deficient IgG1 may require fewer mAbs on the surface of a target cell to effect the binding and subsequent activation of an effector cell.^27^ A recent report has shown that asymmetric afucosylation can affect the ADCC activity.^28^ Here, we correlate the interactions of an asymmetrically afucosylated antibody with specific Fc receptors to ADCC activity mediated by NK cells.

JNJ-61186372 is a low fucose (LF) bispecific antibody (BsAb) generated through controlled Fab arm exchange (cFAE) that targets both EGFR and c-Met with 2 distinct monovalent Fab arms.^29,30^ Multiple mechanisms of action have been attributed to its activity in EGFR-mutant NSCLC. These include signaling inhibition of EGFR and c-Met pathways and Fc effector functions.^18,31^ Here, we define the Fc effector-mediated activity of JNJ-61186372 in multiple EGFR-mutant NSCLC cell lines.

## Results

### Low fucosylation results in enhanced antibody binding to FcγRIIIa

**Figure 1.**
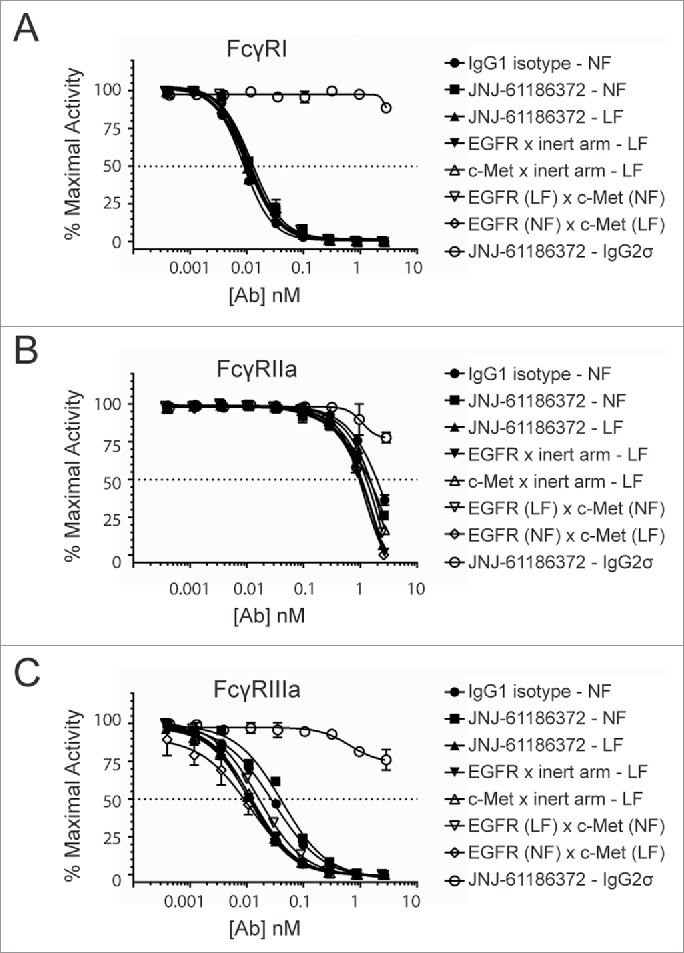
Increased FcγRIIIa binding of low-and hemi-fucosylated forms of JNJ-61186372. FcγRI (A), FcγRIIa (B), and FcγRIIIa (C) binding by antibodies produced in normal fucose (NF) or low fucose (LF) cell line was assessed by competitive Alpha Screen and compared to a wild type IgG1 control antibody (closed circle). JNJ-61186372 – NF (closed square), JNJ-61186372 – LF (closed up triangle), EGFR x inert arm –LF (closed down triangle), c-Met x inert arm – LF (open up triangle), EGFR (LF) x c-Met (NF) (open down triangle), EGFR (NF) x c-Met (LF) (open diamond), JNJ-61186372 – IgG2σ (open circle). Representative data from 3 replicate experiments is shown.

JNJ-61186372 was produced in a low fucose production cell line, resulting in low levels (<10%) of antibodies containing a core fucose attached to the N-linked glycan on Asn297 in the Fc region. The fucose levels of this antibody were determined via liquid chromatography-mass spectrometry, and shown to be reduced by 90% compared to normally fucosylated (NF) mAbs (data not shown). The effect of low fucosylation on antibody binding to FcγRI, FcγRIIa, and FcγRIIIa was evaluated using an AlphaScreen® competitive binding assay. As shown in Fig. 1C, the LF form of JNJ-61186372 (JNJ-61186372-LF) had enhanced binding to FcγRIIIa compared to JNJ-61186372-NF or an IgG1 isotype control antibody. There was no effect of low-level fucosylation on binding to FcγRI and FcγRIIa, as indicated by the identical competition binding curves observed with the LF and NF versions of JNJ-61186372 and with the NF isotype IgG1 antibody (Fig. 1A–B). The controlled Fab arm exchange (cFAE) technology used to generate bispecific antibodies^29^ allowed us to produce hemifucosylated antibodies, where one of the parental antibodies was produced with LF and the other with NF. Hemifucosylated forms of JNJ-61186372 showed enhanced binding to FcγRIIIa, similar to that observed with JNJ-61186372-LF (Fig. 1C). The IC_50_ values of the competition binding profiles are summarized in Table 1. The LF or hemifucosylated versions of JNJ-61186372 showed 2.6- to 3.8-fold higher binding as measured by concentration of antibody required to reach 50% maximal FcγRIIIa binding activity compared to that of JNJ-61186372-NF (Table 2). The hemifucosylated BsAb bound to FcγRI and FcγRIIa similarly to the LF and NF versions of JNJ-61186372. As expected, the Fc-silent IgG2σ version of JNJ-61186372 showed little to no binding to all 3 FcγRs tested (Fig. 1).

**Table 1.** Concentration of antibody (nM) to elicit 50% of maximal FcγR binding activity (IC_50_). Data presented in mean ± SEM of 2–3 independent experiments. *Difference between JNJ-61186372 - LF and JNJ-61186372 – NF is statistically significant (p value = 0.0001).

		FcγRI	FcγRIIa	FcγRIIIa
IgG1 isotype - NF	average	8.7	1269.4	32.4
	± SEM	1.0	340.0	2.9
JNJ-61186372 - LF	average	9.2	946.4	10.0
	± SEM	1.0	119.8	0.6
JNJ-61186372 - NF	average	11.6	1082.3	38.1*
	± SEM	1.4	351.2	0.7
EGFR x inert arm - LF	average	9.3	819.6	10.0*
	± SEM	1.2	138.3	2.0
c-Met x inert arm - LF	average	8.9	1280.0	11.3
	± SEM	1.4	197.6	1.0
EGFR (LF) x c-MET (NF)	average	10.4	975.8	14.6
	± SEM	1.5	296.2	1.6
EGFR (NF) x c-MET (LF)	average	10.8	827.9	11.1
	± SEM	0.8	191.0	1.8

NF = normal levels of core fucosylation; LF = low levels of core fucosylation.

**Table 2.** Fold change to multiple FcγR binding (IC_50_) of different antibodies to that of JNJ-61186372 – NF.

		FcγRI	FcγRIIa	FcγRIIIa
IgG1 isotype - NF	average	1.4	1.0	1.2
	± SEM	0.2	0.2	0.1
JNJ-61186372 - LF	average	1.3	1.2	3.8
	± SEM	0.1	0.2	0.2
JNJ-61186372 - NF	average	1.0	1.1	1.0
	± SEM	0.1	0.4	0.0
EGFR x inert arm - LF	average	1.3	1.4	4.0
	± SEM	0.2	0.2	0.8
c-Met x inert arm - LF	average	1.3	0.9	3.4
	± SEM	0.2	0.1	0.3
EGFR (LF) x c-Met (NF)	average	1.1	1.2	2.7
	± SEM	0.2	0.4	0.3
EGFR (NF) x c-Met (LF)	average	1.1	1.4	3.3
	± SEM	0.1	0.3	0.7

NF = normal levels of core fucosylation; LF = low levels of core fucosylation.

### ADCC activity is enhanced with low fucose versions of JNJ-61186372

**Figure 2.**
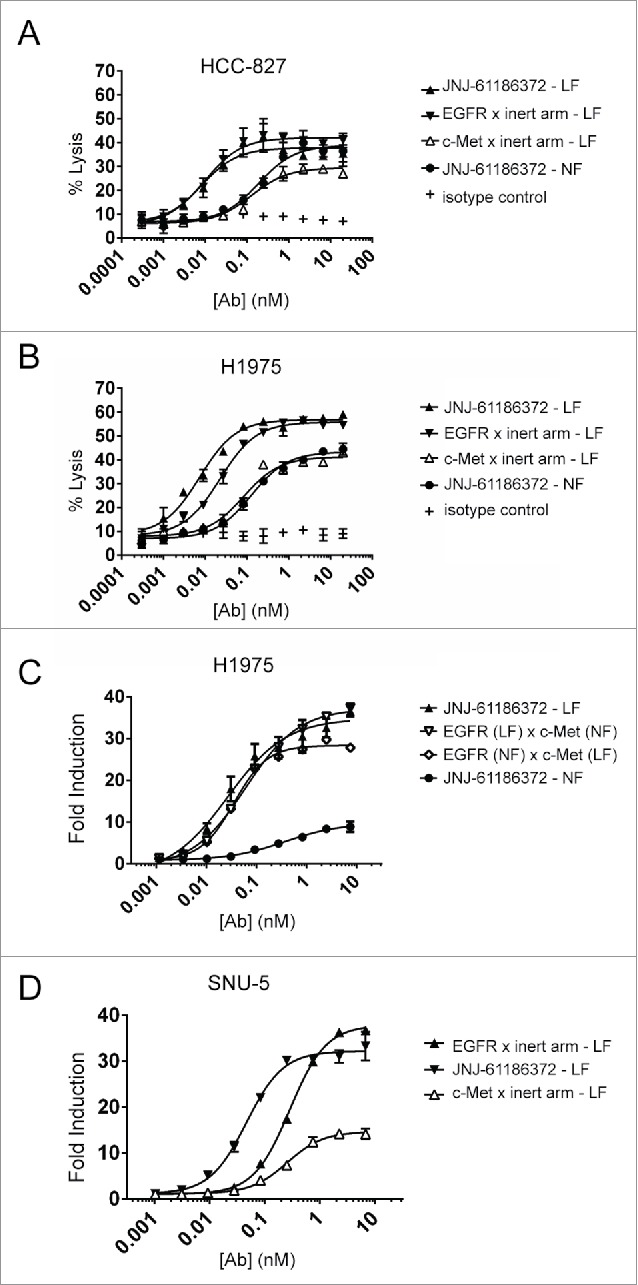
Enhanced ADCC activity of low- and hemi-fucosylated forms of JNJ-61186372. ADCC activity of JNJ-61186372 – LF (closed up triangle), EGFR x inert arm - LF (closed down triangle), c-Met x inert arm – LF (open up triangle), JNJ-61186372 – NF (closed circle), and an IgG1 isotype control (plus sign) was evaluated with donor PBMCs as the source of effector cells against BATDA-labeled HCC-827 (A) and H1975 (B) target NSCLC cells, by Europium detection of dye release after 2 hr. incubation. (C) ADCC activity of JNJ-61186372 – NF (closed circle), JNJ-61186372 – LF (closed up triangle), EGFR (LF) x c-Met (NF) (open down triangle), and EGFR (NF) x c-Met (LF) (open diamond) was evaluated against H1975 using an ADCC surrogate reporter assay. (D) ADCC activity of JNJ-61186372 – LF (closed up triangle), EGFR x inert arm –LF (closed down triangle), and c-Met x inert arm – LF (open up triangle) was evaluated against SNU-5 cells using an ADCC surrogate reporter assay. Representative data from ≥ 3 replicate were shown for each set of experiments.

The NK-mediated ADCC activity of the different fucosylated forms of JNJ-61186372 was evaluated against 2 NSCLC cell lines (H1975 and HCC-827) and compared to that of LF variants of EGFR or c-Met monovalent antibodies. The EGFR and c-Met monovalent antibodies were made of an EGFR- or c-Met-binding arm, respectively, and a non-cellular target binding arm (anti-RSV-F viral surface protein (B21M)). All parental antibodies were generated in LF producing cell lines (low level core fucosylation < 10%). HCC-827 and H1975 were selected based on their differing EGFR mutation status and a varied EGFR: c-Met surface expression ratio (Table 3). In both cell lines, JNJ-61186372-LF was most potent (lowest EC_20_, concentration of antibody needed to achieve 20% lysis) and reached the highest efficacy (E_max_). Monovalent EGFR control antibody (LF) required 1.9- to 3.4-fold higher concentration to reach the EC_20_ value of JNJ-61186372-LF in HCC827 and H1975 cells, respectively, but reached similar E_max_ value to JNJ-61186372-LF. The monovalent c-Met control antibody (LF) required 5- to 35-fold higher concentration to reach the E_20_ value of JNJ-61186372-LF in HCC827 and H1975 cells, respectively, and with a reduced E_max_ value compared to that of JNJ-61186372-LF in both cell lines. Supported by the reduced binding to FcγRIIIa shown in Fig. 1, JNJ-61186372-NF showed reduced ADCC activity against both cell lines compared to JNJ-61186372-LF (17- and 100-fold in HCC827 and H1975, respectively) (Fig. 2A–B, Tables 4-5).

**Table 3.** EGFR and c-Met mutational status in selected cancer cell lines. ACA, adenocarcinoma; del, deletion mutant; WT, wild-type; Y, Yes; N, No. ^29^

				EGFR		c-Met	
Cell Line	Tumor Type	ATCC	EGFR: c-Met Surface ratio	Genotype	Amplified (copies/cell)	Genotype	Amplified (copies/cell)
HCC-827	Lung ACA	CRL-2868	2.0	Del(E746, A750)	Y (19)	WT	N
H1975	Lung ACA	CRL-5908	0.7	L858R, T790M	N	WT	N
SNU-5	Stomach ACA	CRL-5973	0.2	WT	N	WT	Y (9)

**Table 4.** ADCC Activity of antibodies against H1975 lung cancer cell line. Average ± SEM of Emax (% compared to complete lysis) and EC_20_ (the interpolated antibody concentration needed to elicit 20% lysis) for different antibodies.

	E_max_ (%)		EC_20_ (nM)	
	Average	±SEM	Average	±SEM
JNJ-61186372 - LF	59	5.38	0.0015	0.0007
c-Met x inert arm - LF	39	1.12	0.0528	0.0172
EGFR x inert arm - LF	58	4.50	0.0051	0.0012
JNJ-61186372 - NF	40	1.48	0.1505	0.1052

NF = normal levels of core fucosylation; LF = low levels of core fucosylation.

**Table 5.** ADCC activity of antibodies against HCC-827 lung cancer cell line. The average ± SEM of the Emax (% compared to complete lysis) and EC_20_ (the interpolated antibody concentration needed to elicit 20% lysis) for different antibodies.

	E_max_ (%)		EC_20_ (nM)	
	Average	±SEM	Average	±SEM
JNJ-61186372 - LF	31	6.62	0.0376	0.0313
c-Met x inert arm - LF	24	5.30	0.1864	N/A
EGFR x inert arm - LF	30	6.00	0.0708	0.0591
JNJ-61186372 - NF	30	4.59	0.6446	0.2655

NF = normal levels of core fucosylation; LF = low levels of core fucosylation.

The ADCC activity of both hemifucosylated versions of JNJ-61186372 was evaluated using an ADCC surrogate reporter assay in H1975 cells. In support of the FcγRIIIa binding results, both hemifucosylated versions of JNJ-61186372 showed enhanced ADCC activity similar to that observed with JNJ-61186372-LF compared with JNJ-61186372-NF (Fig. 2C). These results support earlier reported findings demonstrating that the absence of core fucose in one half of the antibody Fc was sufficient to enhance both FcγRIIIa binding and ADCC activity of the given antibody.^10,28,^^32^

As shown above in Fig. 2A–B, the c-Met monovalent antibody showed reduced ADCC activity compared to that of the EGFR monovalent antibody. The ADCC activity of antibodies was also evaluated in SNU-5 cells (gastric adenocarcinoma) with high c-Met expression due to gene amplification and consequently with a low EGFR : c-Met surface ratio of 0.2 (Table 3).^31^ Despite this low EGFR: c-Met surface ratio, the ADCC activity of EGFR monovalent antibody still outperformed that of c-Met monovalent antibody (Fig. 2D). Collectively, these results suggest that the ADCC activity of JNJ-61186372 is driven upon binding of the anti-EGFR arm of the BsAb.

### ADCP activity against H1975 and HCC-827 cells is driven upon binding of the anti-EGFR arm of JNJ-61186372

**Figure 3.**
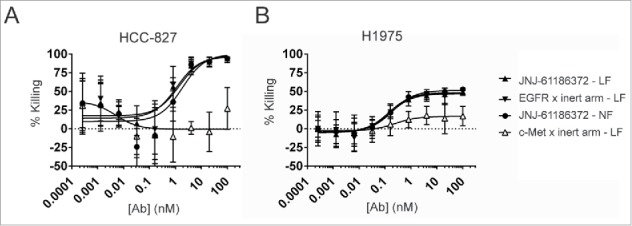
JNJ-61186372 (LF and NF forms) and EGFR monovalent but not c-Met monovalent antibody demonstrated ADCP activity. ADCP activity of JNJ-61186372 – LF (closed up triangle), EGFR x inert arm –LF (closed down triangle), c-Met x inert arm – LF (open up triangle), and JNJ-61186372 – NF (closed circle) was evaluated using in vitro differentiated donor human macrophages as effector cell and GFP positive HCC-827 (A) or H1975 (B) target cells. Macrophages and tumor cells 4 : 1 (E : T) were incubated with serial dilutions of the indicated antibody for 24 hr before detection (GFP) of target cell elimination by flow cytometry. Representative data from ≥ 3 replicate experiments for each target cell line is shown.

The ADCP activity of JNJ-61186372-LF was measured and compared to that of JNJ-61186372-NF, and to that of the monovalent controls for both EGFR or c-Met in H1975 and HCC-827 lines. Statistically comparable ADCP activity was measured for JNJ-61186372-LF, JNJ-61186372-NF, and EGFR monovalent antibodies. The c-Met monovalent antibody showed a markedly reduced ADCP activity (consistently met the statistical criteria for exclusion from the final analyses due to insufficient curve fit) (Fig. 3A–B). Macrophages express FcγRI, FcγRIIa, and FcγRIIIa and can promote ADCP in vitro.^19,33,^^34^ As shown in Fig. 1, JNJ-61186372-LF showed increased binding to only FcγRIIIa. This enhanced FcγRIIIa binding was not sufficient to enhance ADCP activity over JNJ-61186372-NF. Interestingly, similar to what was observed in ADCC assays, the Fc-mediated ADCP activity appeared to be driven by binding of the anti-EGFR arm of the BsAb.

### Fc-mediated effect of JNJ-61186372 through engagement of EGFR binding arm drives in vivo tumor regression of NSCLC human xenograft tumors

Two related in vivo models of NSCLC (H1975 and H1975-HGF) were used to assess the mechanism of action (MOA) of JNJ-61186372. H1975, an EGFR-driven human lung cancer cell line, carries both a primary mutation in EGFR (L858R) and a second site mutation in EGFR (T790M) that confers resistance to small molecule EGFR TKIs such as erlotinib. H1975 cells were further engineered to express human HGF (H1975-HGF) in order to activate the human c-Met pathway following autocrine HGF binding on the tumor cells (mouse HGF does not effectively activate human c-Met).^35^ Thus, H1975-HGF is driven by both EGFR and c-Met pathway activations.

**Figure 4.**
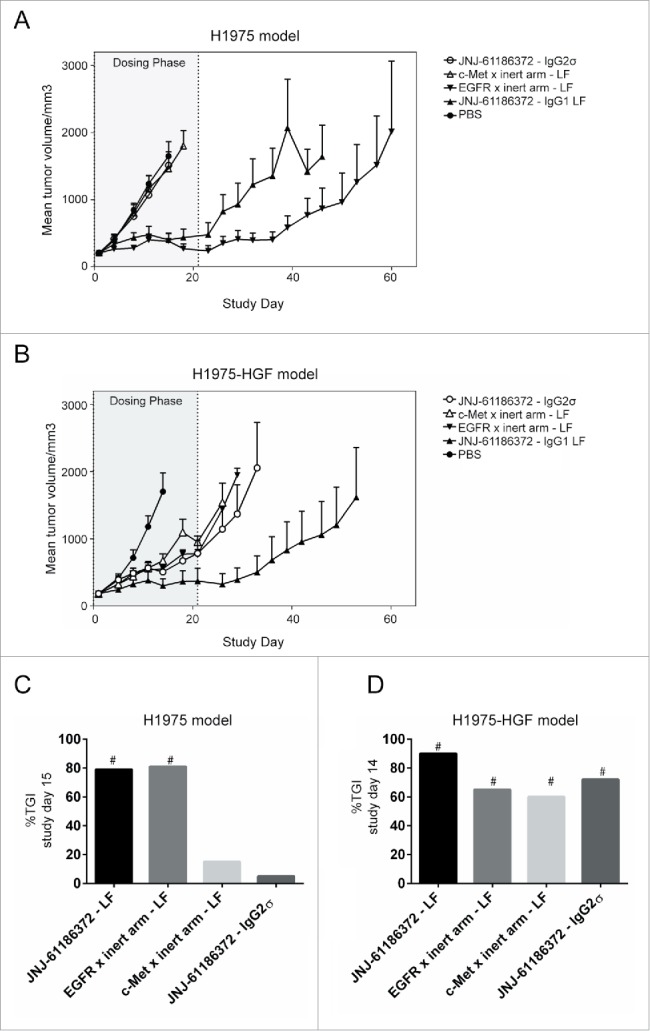
Superior xenograft tumor growth inhibition by JNJ-61186372 produced in low fucose cell line. Female nude mice (n = 8/group) bearing subcutaneous H1975 (A) or H1975-HGF (B) tumors were treated with 10 mg/kg JNJ-61186372 – LF (closed up triangle), EGFR x inert arm – LF (closed down triangle), c-Met x inert arm – LF (open up triangle), JNJ-61186372 – IgG2σ (open circle) or PBS (closed circle) for 21 d (gray shaded dosing phase). Tumor growth was monitored twice weekly until reaching 2000 mm3. Bar graph comparing percent tumor growth inhibition (%TGI) on the day of the final measurement for the PBS control group was compared across all treatments in H1975 (C) and H1975-HGF (D) in vivo study. # indicate multiplicity adjusted Tukey-Kramer p values of ≤ 0.05. The PBS and JNJ-61186372 tumor growth curves in this figure were previously displayed in Moores, et al.^18^

In the first model, H1975 cells were implanted into female nude mice. Tumor-bearing animals were then treated with JNJ-61186372-LF, JNJ-61186372 IgG2σ (Fc-silent), EGFR-LF monovalent, c-Met-LF monovalent, or phosphate-buffered saline (PBS) vehicle control. No significant activity (p > 0.05) was observed with either the c-Met-LF monovalent or JNJ-61186372-IgG2σ antibodies compared to vehicle-treated animals (Fig. 4A). Compared to PBS-treated animals, JNJ-61186372-LF and EGFR-LF monovalent antibodies showed significant percent tumor growth inhibition (% TGI) of 79 and 81, respectively (each with p < 0.05) (Fig. 4C). Both JNJ-61186372-LF and IgG2σ antibodies performed similarly in in vitro phosphorylation assays with similar inhibition of p-Met and p-EGFR (Fig. S1). Therefore, these in vivo results demonstrate the importance of the Fc region in the MOA of JNJ-61186372 efficacy against an EGFR driven NSCLC xenograft model.

In a second tumor model, female nude mice bearing subcutaneous H1975-HGF tumors were treated with the same antibody panels as described above. Unlike the EGFR-driven xenograft study in H1975, partial inhibition of H1975-HGF tumor growth was observed with c-Met-LF monovalent antibody or the IgG2σ version of JNJ-61186372 (Fig. 4D). This inhibition was similar to that observed with EGFR-LF monovalent antibody. However, the strongest tumor growth inhibition was observed with JNJ-61186732-LF (Fig. 4B, D). Thus, efficacy studies using tumor model driven by both EGFR and c-Met revealed that the mechanisms of action of JNJ-671186372 included both inhibition of c-Met and EGFR pathways, as well as Fc effector function mediated upon binding of the anti-EGFR arm of the antibody; all contributed to in vivo efficacy.^18^

### Pharmacodynamic effects of JNJ-61186372 against H1975-HGF lung tumor xenografts

**Figure 5.**
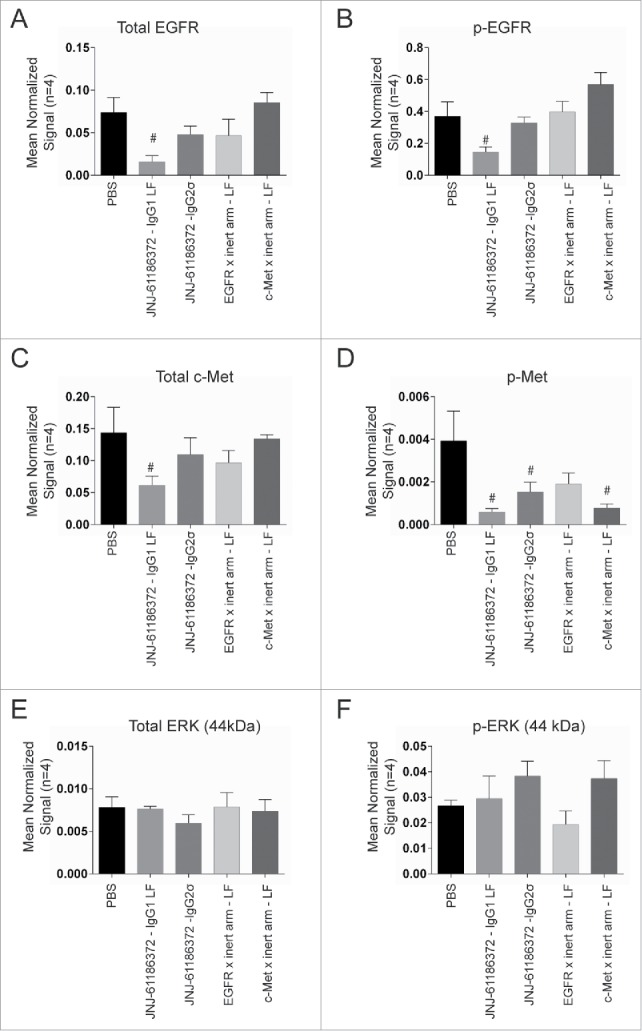
Downmodulation of EGFR and c-Met in H1975-HGF xenograft tumors upon JNJ-61186372 treatment. Female nude mice bearing H1975-HGF lung cancer xenografts were treated with 10 mg/kg of the indicated antibodies or control on study days 1 and 4. Tumors were collected 24 hr after the second dose (day 4). Levels of total EGFR (A), c-Met (C) and ERK1 (p44) (E) and phosphorylated c-Met (pc-MET)(B), pEGFR (D), and pERK1 (p44) (F) were analyzed by Western blot. Graphs show densitometry analysis normalized to actin. # (p < 0.05) indicates statistical significant difference compared to vehicle control (Sidak's multiple comparisons test on normalized signal values).

Twenty-four hours following the second dose (day 4) of JNJ-61186372-LF, H1975-HGF tumors were harvested and pharmacodynamics effects were assessed by Western blot. Levels of total and phosphorylated EGFR, c-Met and ERK1/2 protein in tumor lysates were measured as indicators of receptor down-modulation (total EGFR and c-MET protein) and signaling pathway inhibition (phosphorylated protein). Levels of total and phosphorylated EGFR (p-EGFR) were significantly reduced in tumors from mice treated with JNJ-61186372-LF compared to those treated with vehicle control (Figs. 5A, B). Total c-Met was significantly reduced in tumors from mice treated with JNJ-61186372-LF compared to vehicle control (Fig. 5C). Phosphorylated c-Met (p-Met) was significantly reduced in tumors from mice treated with JNJ-61186372-LF, JNJ-61186372-IgG2σ, and monovalent c-Met-binding antibody compared to vehicle control (Fig. 5D). The 44 and 42 kDa isoforms of ERK (ERK1 and ERK2, respectively) were analyzed individually and no significant treatment-related changes in total or phosphorylated ERK1 (Fig. 5E, F) or ERK2 (data not shown) were measured. Thus, in the H1975-HGF model, while the c-Met arm alone suppressed phosphorylated c-Met, the EGFR arm alone did not demonstrate significant signaling effects. Fc interactions were necessary for down-modulation of both receptors, as tumors from JNJ-61186372-IgG2σ-treated animals did not show a significant decrease in either EGFR or c-Met total receptor levels.

## Discussion

EGFR regulates cellular homeostasis and plays a role in organogenesis and wound healing. Sustained receptor activation occurs during chronic inflammation or upon mutation resulting from carcinogen exposure and oncogenic EGFR is linked to a number of cancer types.^36^ Two classes of EGFR inhibitors, antibody and small molecule drugs, are approved. Anti-EGFR antibodies (e.g., cetuximab, panitumumab, necitumumab) bind to the extracellular receptor, blocking ligand binding leading to receptor internalization and inhibition of sustained receptor activation. Small molecule TKIs (e.g., erlotinib, gefinitib, afatinib) inhibit EGFR autophosphorylation and downstream signaling by competitive inhibition of ATP binding. These two classes of inhibitors are used in largely non-overlapping patient populations. Antibodies (cetuximab, panitumumab) are approved for use in colorectal and head and neck cancers, whereas small molecule TKIs have been approved for treatment of NSCLC, where response rates are strongly associated with the presence of EGFR-activating mutations.^36^ Multiple clinical trials have assessed cetuximab alone and in combination with chemotherapy in NSCLC, but the US Food and Drug Administration and European Medicines Agency rejected approval due to only a small improvement in overall survival benefit coupled with increased side effects, high cost and weekly administration.^37,38^ Another anti-EGFR monoclonal (necitumumab) was recently approved in combination with cisplatin/gemcitabine chemotherapy as a first line treatment in advanced squamous subtype of NSCLC. JNJ-61186372 has been uniquely designed to target both EGFR and c-Met on NSCLC cells through multiple mechanism of action including signaling inhibition of both pathways and activation of Fc effector cells through the EGFR arm.^18^

Resistance to EGFR TKI is common and occurs within the first year of treatment. An EGFR mutation (T790M) is the most frequent cause of resistance to first-generation small molecules in NSCLC.^36^ Another common mechanism of EGFR TKI resistance has been linked to activation of c-Met. Increased c-Met expression in NSCLC is a poor prognostic factor and is found co-expressed in 70% of EGFR-mutant tumors.^39,40^ C-Met amplification occurs in 10–20% of patients with acquired resistance. In TKI-naïve patients, c-Met overexpression can be found in 14–69% of patients.^10,13,^^14,41^ JNJ-61186372 is a low fucose mAb that dually targets EGFR and c-Met and is effective against EGFR-mutated NSCLC cells through multiple mechanisms of action.^18,31^ This dual targeting approach is intended to combat TKI resistance mechanisms either as a follow on therapy or a combinatorial approach with EGFR TKI. The combination approach including JNJ- 61186372 could prevent or delay resistance to EGFR TKI, by acting on multiple clonal populations with varying EGFR mutations within the same tumor, more complete suppression of the EGFR pathway, and by parallel inhibition of c-Met.^16^

The production of JNJ-61186372 in low fucose-generating Chinese hamster ovary (CHO) cells results in an antibody with enhanced binding to FcγRIIIa and enhanced ADCC activity in multiple NSCLC cell lines. By comparing LF, FF, and hemi-fucosylated variations of JNJ-61186372, we confirmed that a lack of core fucosylation in half of the antibody Fc was sufficient to enhance in vitro ADCC activity through enhanced binding to FcγRIIIa. In vitro ADCP activity of JNJ-61186372 was also confirmed and shown to be less effected by the change in Fc core fucosylation because binding to FcγRI and FcγRIIa also mediated ADCP by macrophages. Studies using the single arm EGFR or c-Met monovalent antibodies revealed that the Fc-mediated MOA of JNJ-61186372 was driven through binding of the EGFR arm of the bispecific antibody. This was observed in both ADCC and ADCP experiments where the low fucose monovalent EGFR control performed similar to JNJ-61186372, whereas the monovalent c-Met control antibody had significantly reduced activity in both Fc-mediated immune effector assays. This held true even when the SNU-5 cell line that expressed 5 times more surface c-Met than EGFR was used. Parallel in vivo studies performed in related xenograft models designed to be only EGFR-driven or both EGFR- and c-Met-driven supported the conclusions that the Fc-mediated effect was through the EGFR half of the bispecific antibody. The c-Met monovalent antibody and an Fc-silent IgG2σ version of JNJ-61186372 had no activity in the H1975 (EGFR-driven) model, but had activity in the H1975-HGF (EGFR/c-Met driven), presumably through the effects of signaling inhibition of c-Met pathway in H1975 tumors stimulated by human HGF. Interestingly, despite partial efficacy with JNJ-61186372-IgG2σ, pharmacodynamic assessments of H1975-HGF xenograft tumors highlighted a role for Fc interactions for receptor down-modulation in vivo as no significant change in EGFR or c-Met levels were observed in JNJ-61186372-IgG2σ treated tumors.

An antibody can carry an afucosylated N-glycan in each of the 2 heavy chains. It is likely that most naturally occurring afucosylated glycans exist in antibodies that are hemi-afucosylated, i.e., where one heavy chain carries the afucosylated glycan and the other carries a glycan containing fucose. Crystal structures show that the FcγR binding to IgG is asymmetrical, with both heavy chains making contact with a single FcγRIIIa molecule.^25^ Our results are consistent with structural studies on complexes of afucosylated antibodies with glycosylated FcγRIIIa, whereby the carbohydrate from the receptor interacts with only 1 of the 2 glycans on the Fc. Glycoengineering has been used to manufacture antibodies lacking core fucose, resulting in higher binding affinity to FcγRIIIa and enhanced ADCC activity.^42^ Here, we used cFAE to generate defined hemi-fucosylated antibodies to demonstrate that afucosylation of one of the glycan chains is sufficient to have the desired activity enhancement.^28^ Glycosylation is thought to maintain the Fc in an “open” conformation competent for FcγR binding.^43^ Although glycosylation of one chain can render a partially open state, glycosylation of both chains may be necessary to complete the conformational transition.^44^ Together these observations imply that, although glycosylation of both Fc monomers is critical, afucosylation of half is sufficient to profoundly influence ADCC.^28,32^ Interestingly, fucosylation solely on either the EGFR or c-Met Fc domains of JNJ-61186372 reconfirmed such activity.

Although the low fucose antibodies displayed increased binding to FcγRIIIa, there was little difference in ADCP activity between the normal and low fucose forms of JNJ-61186372. The macrophages used as the effector cell in these ADCP studies express FcγRI, FcγRIIa, FcγRIIb, and FcγRIIIa, all of which can affect ADCP activity.^19,33,^^45^ In the presence of competing IgG, the binding of antibodies engineered to be low fucose was significantly higher than that of their wild-type counterparts, indicating that glycoengineering also enhanced the binding of therapeutic antibodies to myeloid lineage cells as reported in the literature.^22^ Herter and colleagues recently reported that low fucose antibodies had increased binding to monocytes and macrophages.^41^ As reported, glycoengineered antibodies specifically had enhanced affinity to FcγRIIIa, whereas the affinity to FcγRIIa and FcγRI was unaltered.^45^ In their study, the most pronounced effect of enhanced ADCP activity with low fucose antibodies occurred when using IL-10 polarized macrophages (M2c) and in the presence of competing IgG (to block the high affinity FcγRI). The ADCP assay format used in this report did not contain excess IgG (to reduce FcγRI mediated ADCP), which may explain why no difference between the normal and low fucose forms of JNJ-61186372 was observed. It is possible that, in addition to enhanced ADCC, in vivo, in the presence of high concentrations of circulating IgG and dependent on the FcγR expression pattern on surrounding phagoctyes, the ADCP activity of JNJ-61186372 may also be enhanced through increased FcγRIIIa engagement.

Our pharmacodynamics results highlighted a necessity for Fc interactions for downregulation of EGFR and c-Met in the H1975-HGF model. The partial inhibition in tumor growth observed with JNJ-61186372-IgG2σ and the c-Met monovalent antibody was proposed to be due to inhibition of c-Met signaling as the in vitro data and in vivo H1975 model data presented here suggest that the EGFR arm drives the Fc effector mechanism. This was in contrast to a report of an in vivo xenograft model of breast cancer where an Fc- activity deficient form of trastuzumab was able to fully block Her2 and downstream ERK and AKT phosphorylation despite only partial inhibition of tumor growth.^46^ Our result suggested that full inhibition of H1975-HGF tumor growth by JNJ-61186372 required downmodulation of the receptors, and functional Fc binding was necessary for this activity. An alternative explanation was that this decrease might not be due to Fc-activity receptor downregulation, but a selection effect where the antibodies could be binding to cells with high-EGFR/c-Met expression, and the host's NK cells could be destroying those marked cells. Future studies are needed to fully understand this observation.

In vitro and in vivo studies with JNJ-61186372 highlighted that the Fc-mediated MOA occurs through binding of the EGFR arm of the antibody. This was observed in vitro and in vivo with multiple cell lines expressing different levels and ratios of EGFR and c-Met. It is not likely that affinity to the target antigen is a contributing factor because the EGFR arm binds less tightly to EGFR than the c-Met arm binds to c-Met^18,31^ We believe that the epitope played a large role in ADCC activity as reported for anti-CD20 antibodies.^47^ The EGFR arm was based on zalutumumab, which has known ADCC activity.^48,49^ However, the c-Met parental of JNJ-61186372 has weaker ADCC activity and like other bivalent c-Met antibodies is activating. During the discovery of JNJ-61186372, we generated a matrix of monovalent and bivalent EGFR and c-Met binding arms from a panel of anti-EGFR and anti-c-Met antibodies in order to select the best combination for the JNJ-61186372 bispecific antibody. These molecules included different antagonistic and agonistic anti-EGFR and anti-c-Met Abs. For the selection, we eliminated the BsAbs that resulted in loss of binding, increases of lung cancer cell proliferation, and increases in EGFR and c-Met phosphorylation. Empirical evaluations of these molecules using the aforementioned criteria was needed to identify the best pair of anti-EGFR and anti-c-Met Abs to be put into the JNJ-61186372 BsAb design. A recent report of an antagonistic anti-Met antibody with Fc engineering has been described to have enhanced ADCC activity.^50^ This antibody outperformed an ADCC-dead version of the antibody that could still inhibit c-Met signaling in multiple mouse models.^50^

We demonstrated that low level core fucosylation was required on only one half of the BsAb to enhance FcγRIIIa binding and ADCC. Interestingly, studies using monovalent control mAbs for EGFR or c-Met revealed that the Fc activity of JNJ-61186372 was more dependent on the target engagement of the EGFR Fab arm than of the c-Met arm. The findings were confirmed with in vivo studies where the c-Met monovalent antibody and an Fc-silent version of JNJ-61186372 had no activity in an EGFR-driven NSCLC xenograft. However, in a different model that was dependent on c-Met signaling in addition to EGFR signaling, both the Fc-silent JNJ-61186372 and the c-Met monovalent antibody each showed partial inhibition. Interestingly, Fc interactions were important for down-modulation of receptors and inhibition of signaling by JNJ-61186372 since the Fc-silent form of the antibody was not as effective. These activities, in combination with dual signaling inhibition of both the EGFR and c-Met pathways, highlighted multiple mechanisms employed by JNJ-61186372, and supported testing in human clinical trials in NSCLC patients.

## Materials and methods

### Cell culture and engineered cell lines

For these studies, 2 NSCLC tumor cell lines (HCC-827 and H1975) were selected with diversity in the mutational status of EGFR and gene copy number of MET (Table 3). Snu-5 cells (stomach adenocarcinoma) were also selected due to the high level of c-Met and low EGFR : c-Met ratio. Tumor cell lines were obtained from the American Type Culture Collection, (ATCC, Manassas, VA, USA). They were cultured under standard conditions (37°C, 5% CO_2_, 95% humidity) in RPMI 1640 Medium + GlutaMAX™ + 25 mM HEPES (Life Technologies 72400-047), 10% (v/v) heat inactivated fetal bovine serum (Life Technologies 10082), 0.1 mM non-essential amino acids (Life Technologies 11140), and 1 mM sodium pyruvate (Life Technologies 11360). Media were routinely changed 2 to 3 times per week. Subconfluent cell monolayers were passaged using non-enzymatic cell dissociation buffer (Life Technologies 13151-014).

The H1975-HGF cell line is a human adenocarcinoma cell line (ATCC #CRL-5908) that has been transduced with a lentivirus to express the human HGF gene. Human HGF plasmid was obtained from Genecopoeia (Cat#Ex-A1330-LV105), and HGF lentivirus was generated in-house using the Genecopoeia packaging kit (Cat#STK300-10). The day before the infection, H1975 cells were plated at one million cells in a 100 mm^2^ dish in RPMI media as described above. The cells were allowed to incubate overnight at 37°C and 5% CO_2_. The next day the spent media was removed. One mL of HGF lentivirus was added to 10 mL of media and 10 μg/mL of polybrene). (Polybrene, Millipore, Cat#TR-1003-G, stock = 10 mg/mL) The H1975 cells were allowed to incubate overnight at 37°C and 5% CO_2_, not allowing this incubation with virus to exceed 16 hrs. Early the next day, the media was removed and replaced with media containing 2 μg/mL of puromycin (Invitrogen, Cat# A11138-03). The cells that were alive after 5 d were considered H1975-HGF pooled cells. (A puromycin kill curve was previously performed to determine the optimal concentration that killed 100% of H1975 cells at 2 days). An HGF MSD Assay confirmed secretion of HGF into conditioned media from H1975-HGF cells (data not shown).

H1975 and HCC-827 cells were stably infected with green fluorescent protein (GFP) following sequential spinfections with commercially available pLKO.1-TurboGFP lentiviral particles. Cells were seeded at 0.1 × 10^6^ cells/mL and allowed to adhere overnight to a 6-well tissue culture plate. On the morning of infection, the media was changed, and 2 µg/mL polybrene and 20 µl virus/well was added. The plates were centrifuged at 525 g for 60 min at room temperature. After the spin, the media was aspirated and fresh media plus virus/polybrene added for a second centrifugation. The cells rested for 24–48 hours before being selected with 2 µg/mL puromycin until a stable bright GFP+ population was generated. GFP expression was verified by flow cytometry and the cells were banked and cryopreserved. Cells were maintained in culture with media containing puromycin.

### Preparation of parental mAbs

Parental mAbs used to generate the EGFR x c-Met bispecific antibody (JNJ-61186372) were prepared from CHO cell lines that incorporate very low levels of fucose into mAbs. Using the low fucose host cell line, standard conditions were used to generate the respective stable cell lines for the parental EGFR F405L and c-Met K409R mAbs.^30^ The cell lines for the respective parental EGFR F405L and c-Met K409R mAbs were grown in conditioned media and harvested for purification after 6 d. The parental mAbs were purified from the harvested clarified media using MabSelect SuRe resin (GE Healthcare, Cat# 17-5438-03). The purity of the antibodies was analyzed by SDS–10% polyacrylamide gel electrophoresis (PAGE). After neutralization into Tris pH 7.2, the solutions were dialyzed into dDPBS, then 293SFMII production media (Invitrogen Cat# 11686029) 24 h later.

### Controlled Fab-arm Exchange (cFAE) to generate bispecific Abs

Bispecific human IgG_1_ mAbs are produced from the 2 purified bivalent parental antibodies each of which has been modified to carry a single complementary matched mutation in its third constant domain (CH3): K409R and F405L.^30^ Briefly, the 2 parental mAbs, IgG1-F405L and IgG1-K409R, are mixed in equimolar amounts and allowed to undergo recombination at 31°C for 5 hours in the presence of 75 mM of the mild reducing agent 2-mercaptoethylamine-HCl (2-MEA, Sigma-Aldrich, cat no. 30070) as previously described.^12^ The 2-MEA is removed by 3 rounds of PBS dialysis using a Slide-A-Lyzer cassette (Thermo Scientific) as per the manufacturer's instructions. The dialyzed solution is passed through a low protein binding 0.2 µm syringe filter (Pall Corporation) in a sterile flow-cabinet. Bispecific quality is assessed by SDS/PAGE, HP-SEC and hydrophobic interaction liquid chromatography (HIC). Bispecific yields are typically > 95%.

### Binding to Fc receptors

Competitive binding of IgGs to FcγRs was assessed by AlphaScreen™ (PerkinElmer), a bead-based, homogeneous proximity assay platform.^26^ Human IgG1, biotinylated using a SureLINK™ Chromophoric Biotin Labeling Kit (KPL, cat. no. 86-00-01), was captured on streptavidin donor beads (PerkinElmer, cat. no.6760002). His-tagged FcγRI (R&D Systems, cat no. 1257-FC), FcγRIIa (R&D Systems, cat no. 1330-CD/CF) or FcγRIIIa (R&D Systems, cat no. 4325-FC) were captured on nickel chelate acceptor beads (PerkinElmer, cat. no. CUSM0220400EA). When biotinylated IgG is bound to the His-tagged FcγR, donor bead excitation at 680 nm induces the release of singlet oxygen molecules that excite the acceptor bead, resulting in a chemiluminescent emission at 520–620 nm. Competition by unlabeled competitor antibodies results in a reduction in the maximal signal. Reagents were added in a 50 μL final volume to 96-well ½ area plates (Costar, cat. no. 3693) in the following order and final concentration: Biotinylated human IgG1 (200 ng/mL), unlabeled competing antibodies (applied as serial dilutions, at concentrations specified in Fig. 1), FcγRs (200 ng/mL), nickel chelate acceptor beads (20 μg/mL), and streptavidin donor beads (20 μg/mL). All reagents were diluted in 1xPBS, 0.05% (w/v) bovine serum albumin, 0.01% (w/v) Tween 20, pH 7.2. Plates were sealed with black light absorbing film (USA Scientific, cat. no. 2925–0500), shaken at RT for 1 h, and read on an EnVision® Multilabel Reader (PerkinElmer). Results are reported as the % Maximal Activity per plate, where % Maximal Signal = (Experimental – Minimum)/ (Maximum – Minimum). A value of 100% maximal activity indicates the absence of competition. A four-parameter logistic (4PL) curve fitting model was applied in GraphPad Prism® 6 (GraphPad Software, Inc.) to the log_10_ of the competitor antibody concentration versus the % relative maximal activity. Competitor antibody concentrations at 50% maximal activity were interpolated in Prism® 6 and reported as mean IC50 ± SEM for 2–3 independent experiments.

### ADCC assays

Human peripheral blood mononuclear cells (PBMCs) were isolated from normal donor leukopaks (Biological Specialty Corporation, cat.no. 213-15-04) for use as effector cells and were cryopreserved. For the ADCC experiments described in this report, the same PBMC donor was used for all experiments (heterozygous for both FcγRIIa genotype R131/H131 and FcγRIIIa genotype F158/V158). PBMCs were thawed the day before assay set up and rested overnight in XVIVO-10 (Lonza, cat.no. 04-380Q) plus 10% (v/v) FBS (Gibco, cat.no 10082). On the day of set up, tumor cells were labeled with BADTA reagent (Perkin Elmer Life Sciences, cat.no. C136-100) for 30 min under standard conditions (37°C 5% CO_2_, 95% humidity), washed twice, and re-suspended in respective tumor cell mediums described above. PBMCs and tumor cells were combined at a 25:1 effector to target cell ratio (0.25 × 10^6^ effector cells: 1 × 10^4^ target cells) along with serial dilutions of test antibody in 200 µL total in 96-well U-bottom plates. Plates were centrifuged at 200 g for 2 min to facilitate cell-to-cell contacts then incubated at standard conditions for 2–3 hours. Following incubation, assay plates were centrifuged at 200 g for 5 min and 20 µL of supernatant was removed and combined with 200 µL of a Europium solution (Perkin Elmer Life Sciences, cat.no. C135-100). Plates were incubated in the dark with shaking for 15 min, then time resolved fluorescence was measured on the Envision 2101 Multilabel Reader (PerkinElmer Life Sciences, cat.no. 2104-0010). Percent lysis was calculated as (Experimental release – Spontaneous release) / (Maximal release – Spontaneous release) X 100. Data were log transformed and fit to a sigmoidal dose response curve using GraphPad Prism. The concentration needed to reach 20% lysis (EC_20_) was determined for each antibody by interpolating from the dose response curve. The top of the curve (E_max_) was determined to identify the maximal lysis for each antibody.

### Surrogate ADCC assay

The surrogate assay was performed using the ADCC Reporter Bioassay Core Kit (Promega, cat no. G7018). Optimal Effector : Target (E : T) cell ratios were determined to be ∼6 : 1. H1975 were harvested, washed twice with dPBS and resuspended at 5 × 10^5^ cells/mL. Twenty-five μL/well (12.5 k cells/well) of cell suspension was plated in 96-well, white, flat-bottomed plates (Corning®, cat no. 3917). Perimeter wells received buffer only. Plates were placed in a CO_2_ incubator at 37°C for 20–24 h before the assay. Suspension cells (SNU-5) were plated identically, only without an overnight incubation. Next, serial dilutions of test antibodies were prepared in prewarmed ADCC assay buffer (RPMI1640 + Low IgG serum), and 25 μL/well was added to plates, followed by gentle mixing. Then, 25 μL/well (75 k cells/well) of freshly thawed ADCC Bioassay Effector Cells, diluted to 3 × 10^6^ cells/well in ADCC assay buffer were added to each well. Plates were covered with a lid and incubated in a 37°C incubator for 6 h. Assay plates were equilibrated to RT for 15 min. 75 μL/well of Bio-Glo Luciferase Assay Reagent was added to the inner 60 wells of the assay plate, as well as to 3 outer wells for background determination. Plates were covered with foil and incubated at RT for 15 min. Luminescence was recorded in relative light units (RLU) on an EnVision® Multilabel Reader (PerkinElmer). The Fold of Induction was calculated using the following formula: RLU (induced–background) /RLU (no antibody control–background). Data was plotted in GraphPad Prism® 6 (GraphPad Software, Inc.) as Log_10_ [antibody] vs. RLUs and Log_10_ [antibody] versus Fold of Induction. Curve-fitting was performed in Prism® 6 using a 4 parameter logistic (4PL) model.

### In vitro macrophage differentiation

CD14+ monocytes were purified from PBMCs by negative depletion using a CD14 isolation kit without CD16 depletion. Monocytes were plated at 0.1 × 10^6^ cells/cm^2^ in X-VIVO-10 medium containing 10% (v/v) FBS and differentiated to macrophages with 25 ng/mL M-CSF (R&D Systems 216-MC/CF) for 7 d. Fifty ng/mL of interferon γ (R&D Systems 285-IF/CF) was added for the final 24 hours of differentiation to polarize to M1 macrophages. For the experiments described here, 2 different PBMC donors were used (heterozygous for both FcγRIIa genotype R131/H131 and FcγRIIIa genotype F158/V158). Genotyping was performed via genomic DNA PCR as a commercial service by Assuragen.

### 24 hr ADCP

Isolated macrophages were incubated with GFP+ NSCLC cells at a ratio of 4:1 (0.1 × 10^6^ macrophages: 0.025 × 10^6^ target cells) for 24 hr with or without mAbs in 96-well U-bottom plates in target cell culture media. After incubation, cells were removed from the 96-well plates using Accutase. Macrophages were identified with anti-CD11b and anti-CD14 antibodies (both from BD Biosciences) coupled to AlexaFluor 647 following kit protocol for antibody labeling. Cells were acquired on a BD LSR Fortessa. The data were analyzed using FlowJo Software (Tree Star). Singlet gating was done to remove doublets (2 cells stuck together instead of phagocytosis), and then separate populations of cells were identified using quadrant gating for FITC and AlexaFluor 647. Percent killing was determined by the following equation: =100*((average %FITC+AF647- of [lowest mAb] for each antibody) - %FITC+AF647-sample) / (average %FITC+AF647- of [lowest mAb] for each antibody).

### In vivo tumor models

Animal studies were performed at Charles River Discovery Services (Morrisville, NC). All of the procedures relating to animal care, handling, and treatment in this article were performed according to the guidelines approved by Institutional Animal Care and Use Committee. Briefly, 5 × 10^6^ H1975 or H1975-HGF cells were injected subcutaneously into the flanks (injection volume 0.2 mL/mouse) of female nude mice (CD-1 NU/NU, 4–6 weeks old, Charles River Laboratories, Wilmington, MA). When tumor volumes reached a mean size of ∼200 mm^3^, animals were treated with antibodies (10 mg/kg, dosed 2X/week intraperitoneally, 8 animals per treatment group). Tumor size measured on 18 different days within the 60 day study. Groups were terminated when the mean tumor volume (MTV) of the group reached 1500–2000 mm^3^. Data are presented as mean + SEM. The percent tumor growth inhibition (%TGI) for group A vs group B (PBS vehicle group) comparison was calculated as (1-A/B) × 100%, where A and B are the least squares mean estimates of tumor volume of treatments A and B, respectively, on the day the PBS control group reached endpoint (day 15 for H1975 study; day 14 for H1975-HGF study). Statistical analysis was completed using log transformed tumor volume data and a Tukey-Kramer p value adjustment for multiplicity was included.

Tumors were collected and snap frozen 24 hours after the day 4 dose of antibody and shipped to Janssen for analysis by Western blot. Frozen tumors were weighed, cut into pieces and transferred to Lysing Matrix A tubes (MP Biomedicals 6910–100) on dry ice. Ice cold RIPA buffer (Thermo 89901) containing 1 mM EDTA (Invitrogen 15575-038), 1 mM EGTA (BioWorld 40520008-2), 10% (w/v) glycerol, 2X concentrated HALT phosphatase inhibitor solution (Thermo 78427), 2X concentrated Phosphatase Inhibitor Set 1 (Calbiochem 524624), 2X concentrated Phosphatase Inhibitor Set 2 (Calbiochem 524625), Complete mini EDTA-free protease inhibitor, (Roche 11 836 170 001) and 2 mM PMSF (Sigma-Aldrich 93482-50ML-F) was added to the tubes, and the samples were homogenized using a FastPrep-24 instrument (MP Biomedicals). The lysates were clarified by centrifugation and total protein concentrations were determined using a BCA Protein Assay kit (Pierce 23225) according to the manufacturer's instructions. The standard curve was plotted and the lysate protein concentrations were calculated using SoftMax Pro 6 software associated with the SpectraMax plate reader (Molecular Devices). Remaining tumor lysates were frozen at −80°C until Western blot analysis.

Tumor lysates were thawed on ice and samples were prepared for electrophoresis by mixing 50 ug aliquots of total lysate protein with NuPAGE sample buffer (Invitrogen NP00007) containing NuPAGE reducing agent (Invitrogen NP0004), followed by heating at 70°C for 10 min. The samples were resolved on 4–12% NuPAGE Bis-Tris polyacrylamide gels (Invitrogen NP0335) under reducing conditions. After transfer of the proteins to nitrocellulose membranes (Invitrogen LC2001), the membranes were blocked with Odyssey Blocking Buffer (LI-COR 927-4000) for 1 hour at room temperature, and then incubated with the following antibodies overnight at 4°C: mouse monoclonal anti-human EGFR (EGF-R2; Santa Cruz Biotechnology sc-73511), rabbit polyclonal anti-human EGFR [pY^1173^] (Invitrogen 44-794G), mouse monoclonal anti-human Met (L41G3; Cell Signaling Technology 3148), rabbit monoclonal anti-human phospho-Met [Tyr1234/1235] (D26; Cell Signaling Technology 3077), mouse monoclonal anti-human MAP kinase (Invitrogen13-6200), rabbit polyclonal anti-human phospho-ERK1+2(Thr202/Tyr204) (Invitrogen36-8800), and goat polyclonal anti-human actin (C-11; Santa Cruz Biotechnology sc-1615). Following the overnight incubation, the membranes were washed several times with TBS/ 0.05% (w/v) Tween-20 (Thermo T9039), and then incubated with the following secondary antibodies for 1 hour at room temperature in the dark: donkey anti-rabbit IR Dye 800, donkey anti-goat IR Dye 680, and donkey anti-mouse IR Dye 680 (LI-COR 926-32213, 926-32224, 926-32222, respectively). The membranes were again washed several times, rinsed briefly with PBS, and scanned on the Odyssey Imaging System (LI-COR). Densitometry analysis to quantify the levels of total and phosphorylated EGFR, c-Met, ERK1, and actin protein was performed using Odyssey Image Studio Version 2.0 software (LI-COR). The band densities of the target proteins were normalized relative to actin. The normalized data was analyzed using GraphPad Prism 6 software. Ordinary one-way ANOVA and 2-sided, unpaired Sidak's multiple comparisons tests were used to compare treatment groups. For data sets with unequal variances, log transformation was performed prior to final analysis. p-values less than 0.05 were considered significant.

## Supplementary Material

Supplemental_Data.zip
