# Supplementary material for: Fc-mediated activity of EGFR x c-Met bispecific antibody JNJ-61186372 enhanced killing of lung cancer cells
Source: MAbs. 2016 Oct 27;9(1):114–26. doi: 10.1080/19420862.2016.1249079 (PMC5240640; doi:10.1080/19420862.2016.1249079)
Supplement: Supplemental_Data.zip [file kmab-09-01-1249079-s001.zip › 1. Supplemental Figure Legend and Method.docx]

**Supplemental Figure Legend and Method**

**Figure S1**

H1975 cells were serum-starved overnight, then treated with indicated antibodies for 1 hour, followed by ligand stimulation for 15 minutes. Cells were lysed and phospho-EGFR(Tyr1173) (A) and phospho-c-Met (Tyr1349) (B) levels measured using a Meso Scale Discovery (MSD) assay. Error bars indicate SEM of triplicate samples. IC_50_ (nM) values were calculated using GraphPad Prism 6 software. Dashed line indicates ligand treated control level of pEGFR or pMet. Dotted line indicates untreated control level.

**In vitro phosphorylation assays**

H1975 cells were incubated overnight to adhere to a flat bottom 96-well assay plate at 37^o^C, 5% CO_2_ before being serum-starved for 24 hr. On the day of the assay, the cells were pre-treated with serial dilutions of JNJ-61186372-LF and JNJ-61186372-IgG2σ for 1 hr prior to 15 min stimulation with 100 ng/mL HGF or 50 ng/mL EGF. Upon completion of treatment incubation, the levels of phosphorylated c-Met (pMet) and phosphorylated EGFR (pEGFR) were determined using Phospho-Met (Tyr1349) Assay Whole Cell Lysate MSD Kit (Meso Scale Discovery) and Phospho-EGFR (Tyr1173) Assay Whole Cell Lysate MSD Kit (Meso Scale Discovery) according to the manufacturer’s instructions. Briefly, the treatment medium was removed, and the cells were lysed in 70 uL/well MSD complete lysis buffer. After incubation for 10 minutes on a plate shaker at room temperature, 30 uL aliquots of the cell lysates were transferred to previously blocked/washed MSD assay plates configured for measuring either pMet or pEGFR. Following incubation for 1 hour on a plate shaker at room temperature, the lysates were decanted, the plates were washed, and analyte-specific detection antibody solutions were applied. The plates were incubated under foil plate covers for 1 hour on a plate shaker at room temperature and then washed. MSD Read Buffer was added and electrochemiluminescent (ECL) signals were recorded on a SECTOR Imager 6000 (Meso Scale Discovery) instrument using standard 96-well detection parameters. Data were plotted as the logarithm of antibody concentration versus ECL signal. IC_50_ values were determined by fitting the data to a 4-parameter (variable slope) sigmoid inhibition model using GraphPad Prism 6 software.
