# Supplementary material for: Fc-mediated activity of EGFR x c-Met bispecific antibody JNJ-61186372 enhanced killing of lung cancer cells
Source: MAbs. 2016 Oct 27;9(1):114–26. doi: 10.1080/19420862.2016.1249079 (PMC5240640; doi:10.1080/19420862.2016.1249079)
Supplement: Supplemental_Data.zip [file kmab-09-01-1249079-s001.zip › 3. Supplemental Table 1-10.pdf]

Supplemental File
Tables of raw data graphed in Figures 1-4

Data presented in the figures and supplemental tables is representative data from one of the indicated number of experiments as detailed in each figure legend.
Data presented inTables 1, 2, 4, & 5 is compiled data with standard error from multiple experiments

Table S1 - Figure 1A FcgammaRI

[Ab] (nM)	IgG1 isotype - NF	JNJ-61186372 - LF	EGFR x inert arm - LF	c-Met x inert arm - LF	EGFR (LF) x c-Met (NF)	EGFR (NF) x c-Met (LF)	JNJ-61186372 - NF	JNJ-61186372 - IgG2sigma
0.0	98.2	97.465	97.48	96.76	100	96.92		94.325
0.0							97.475	
0.4							99.685	
0.4	99.12	99.245	100	98.37	99.68	99.735		97
1.1		100			99.63			
1.2							100	
1.2	99.535		99.115	100		99.125		
1.3								97.3
3.3		95.67			97.505			
3.5						93.735		
3.6							96.305	
3.7	83.905		89.055	88.075				
3.9								97.285
9.9					52.76			
10.0		48.25						
10.6						50.82		
10.7							53.335	
11.0	40.285		43.855	41.645				
11.7								99.28
29.6					22.175			
30.1		20.985						
31.9						17.725		
32.1							22.54	
32.9	12.155		18.87	22.34				
35.2								95.85
88.9					7.77			
90.4		6.74						
95.8						5.225		
96.3							8.575	
98.8	3.06		5.005	4.98				
105.7								95.82
266.7					1.625			
271.1		1.31						
287.4						1.175		
288.9							1.83	
296.3	0.705		0.68	0.87				
317.0								99.865
800.0					0.325			
813.3		0.23						
862.2						0.225		
866.7							0.26	
888.9	0.14		0.1	0.11				
951.1								97.66
2400.0					0.06			
2440.0		0.14						
2586.7						0.06		
2600.0							0.005	
2666.7	0.01		0.015	0.03				
2853.3								88.73

Table S2 - Figure 1B FcgammaRIIa

[Ab] (nM)	IgG1 isotype - NF	JNJ-61186372 - LF	EGFR x inert arm - LF	c-Met x inert arm - LF	EGFR (LF) x c-Met (NF)	EGFR (NF) x c-Met (LF)	JNJ-61186372 - NF	JNJ-61186372 - IgG2sigma
0	97.145	96.38	98.135	95.75	97.11	97.445	97.2	95.545
0.365798	99.31	97.225	97.4	97.39	98.42	97.58	98.11	98.085
1.097394		98.26			98.415			
1.182747	99.74		97.4	99.505		97.29	99.245	
1.304679								98.69
3.292181		98.91			100			
3.54824						98.365		
3.566529							99.65	
3.657979	98.425		97.79	98.905				
3.914037								98.09
9.876543					98.93			
10.04115		99.21						
10.64472						99.29		
10.69959							99.77	
10.97394	98.615		99.245	99.035				
11.74211								99.45
29.62963					98.175			
30.12346		97.81						
31.93416						97.775		
32.09877							98.035	
32.92181	99.33		97.6	98.45				
35.22634								97.315
88.88889					96.295			
90.37037		95.305						
95.80247						93.66		
96.2963							91.975	
98.76543	97.015		97.505	97.765				
105.679								98.36
266.6667					89.505			
271.1111		88.46						
287.4074						86.02		
288.8889							85.97	
296.2963	92.745		89.67	90.52				
317.037								97.985
800					67.88			
813.3333		61.06						
862.2222						56.94		
866.6667							65.03	
888.8889	75.86		53.965	70.245				
951.1111								89.83
2400					14.995			
2440		6.51						
2586.667						0.24		
2600							26.175	
2666.667	36.535		1.88	16.935				
2853.333								77.825

Table S3 - Figure 1C FcgammaRIIIa

[Ab] (nM)	IgG1 isotype - NF	JNJ-61186372 - LF	EGFR x inert arm - LF	c-Met x inert arm - LF	EGFR (LF) x c-Met (NF)	EGFR (NF) x c-Met (LF)	JNJ-61186372 - NF	JNJ-61186372 - IgG2sigma
0.0	93.95	100	100	100	96.875	100	98.59	96.575
0.4	100	96.265	95.74	98.555	100	89.515	100	99.91
1.1		90.4			96.585			
1.2	93.18		94.76	90.01		79.115	95.38	
1.3								99.385
3.3		80.685			86.205			
3.5						69.305		
3.6							95.71	
3.7	86.33		80.79	79.44				
3.9								95.76
9.9					63.745			
10.0		51.55						
10.6						46.485		
10.7							73.57	
11.0	70.605		51.145	53.635				
11.7								97.17
29.6					34.545			
30.1		23.83						
31.9						23.49		
32.1							61.64	
32.9	46.945		24.3	24.845				
35.2								95.91
88.9					13.235			
90.4		8.01						
95.8						7.49		
96.3							24.09	
98.8	19.86		6.825	7.83				
105.7								94.83
266.7					2.49			
271.1		1.18						
287.4						1.49		
288.9							8.61	
296.3	5.49		1.025	1.21				
317.0								93.045
800.0					0.17			
813.3		0.04						
862.2						0.21		
866.7							0.94	
888.9	0.15		0.07	0.065				
951.1								81.5
2400.0					0.17			
2440.0		0.015						
2586.7						0.11		
2600.0							0.08	
2666.7	-0.435		0.01	0.03				
2853.3								76.075

Table S4 - Fig 2A

HCC-827

[Ab] (nM)	JNJ-61186372 - LF	c-Met x inert arm - LF	EGFR x inert arm - LF	JNJ-61186372 - NF	IgG1 isotype - NF
20	35.5	27.0	41.0	36.5	7.0
6.7	34.5	29.0	39.5	36.5	7.5
2.2	34.5	29.0	41.0	40.0	8.0
0.7	37.0	27.0	42.0	34.0	9.0
0.2	42.5	24.5	43.0	23.0	9.0
0.08	40.0	12.0	40.5	16.0	10.0
0.03	31.0	9.5	33.0	12.0	9.5
0.009	21.0	9.0	21.5	9.0	8.5
0.003	14.0	6.5	15.0	8.0	8.0
0.001	8.5	6.0	9.0	5.0	6.5
0.0003	8.0	7.0	8.5	7.5	9.5

Tables S5 - Fig 2B

H1975

[Ab] (nM)	JNJ-61186372 - LF	c-Met x inert arm - LF	EGFR x inert arm - LF	JNJ-61186372 - NF	IgG1 isotype - NF
20	59.0	43.0	54.5	44.5	9.0
6.7	57.5	39.0	54.5	43.5	8.5
2.2	57.0	39.0	56.5	40.0	10.5
0.7	53.5	36.0	55.5	36.5	9.5
0.2	56.0	38.0	51.5	31.0	8.0
0.08	54.0	22.5	46.5	21.5	8.0
0.03	43.5	14.5	33.0	13.5	9.5
0.009	33.5	11.5	21.0	11.0	9.0
0.003	26.5	10.0	16.0	9.5	9.0
0.001	15.5	10.0	11.0	6.5	7.5
0.0003	8.0	6.5	7.0	4.5	7.5

Tables S6 - Fig. 2C

[Ab] (nM)	JNJ-61186372 - LF	EGFR (LF) x c-Met (NF)	EGFR (NF) x c-Met (LF)	JNJ-61186372 - NF
7.5	36.6	37.3	27.8	8.9
2.5	32.6	35.2	29.8	8.4
0.8	30.5	32.3	27.4	6.4
0.3	27.8	27.9	25.7	4.8
0.09	25.8	22.8	22.8	3.4
0.03	17.9	13.1	13.2	1.7
0.01	8.4	5.5	5.2	1.2
0.003	3.2	2.2	2.0	1.1
0.001	1.4	1.3	1.3	1.0

Table S7 - Figure 3A

[Ab] (nM)	JNJ-61186372 - LF	EGFR x inert arm - LF	c-Met x inert arm - LF	JNJ-61186372 - NF
101.1	94.2	92.5	27.1	94.0
20.2	89.5	89.0	-4.0	89.6
4.0	84.1	86.9	1.2	85.9
0.8	56.2	59.4	-10.5	35.8
0.2	-9.0	-0.5	-8.1	-9.3
0.03	7.1	-12.1	-10.1	-24.2
0.006	20.5	17.2	21.9	20.0
0.001	32.1	40.3	33.3	31.0
0.0003	34.8	28.4	31.0	34.4

Tables S8 - Figure 3B

[Ab] (nM)	JNJ-61186372 - LF	EGFR x inert arm - LF	c-Met x inert arm - LF	JNJ-61186372 - NF
101.1	49.5	49.1	17.0	52.8
20.2	48.7	43.3	13.8	49.7
4.0	44.5	44.5	17.8	46.9
0.8	39.2	40.3	12.3	42.6
0.2	24.6	22.2	5.9	21.4
0.03	5.6	3.4	-3.0	1.6
0.006	-4.6	-12.0	-5.7	-5.9
0.001	-7.3	-1.5	-0.4	-3.9
0.0003	0.0	0.0	0.0	0.0

Tables S9 - Figure 4A

	PBS		JNJ-61186372 - LF		EGFR x inert arm - LF		c-Met x inert arm - LF		JNJ-61186372 - IgG2sigma	
day	MTV	n	MTV	n	MTV	n	MTV	n	MTV	n
1	206.8	8	206.6	8	206.4	8	207.6	8	206.5	8
4	388.5	8	338.4	8	265.4	8	415.1	8	417.6	8
8	849.1	8	435.6	8	281.6	8	803.8	8	753.1	8
11	1236.8	8	481.3	8	400.4	8	1165.0	8	1071.5	8
15	1650.8	8	403.5	8	384.8	8	1462.6	8	1512.8	8
18			435.9	8	271.1	8	1803.4	8		
23			479.6	8	238.5	8				
26			827.5	8	352.4	8				
29			930.3	8	413.5	8				
32			1226.1	8	396.6	8				
36			1353.3	7	404.1	8				
39			2071.6	7	585.1	8				
43			1420.3	7	770.5	8				
46			1643.0	7	870.1	8				
50					961.3	8				
53					1265.1	8				
57					1518.6	8				
60					2016.9	8				

Tables S10 - Figure 4B

	PBS		JNJ-61186372 - LF		EGFR x inert arm - LF		c-Met x inert arm - LF		JNJ-61186372 - IgG2sigma	
day	MTV	n	MTV	n	MTV	n	MTV	n	MTV	n
1	183.9	8	183.9	8	182.6	8	183.8	8	184.4	8
5	417.3	8	245.4	8	325.6	8	325.1	8	391.8	8
8	718.9	8	328.0	8	483.4	8	442.4	8	489.5	8
11	1182.5	8	381.0	8	534.8	8	565.3	8	566.0	8
14	1702.5	8	301.6	8	555.9	8	675.8	8	508.8	8
18			368.3	8	774.6	8	1099.3	8	674.4	8
21			372.1	8	776.6	8	953.5	8	785.5	8
26			326.5	8	1438.0	8	1538.5	8	1146.9	8
29			393.5	8	1948.0	8			1370.3	8
33			504.3	8					2054.5	8
36			685.9	8						
39			832.8	8						
42			956.9	8						
46			1062.1	8						
49			1207.0	8						
53			1616.8	8						
56										
60										
