# Supplementary figures and images for: Fc-mediated activity of EGFR x c-Met bispecific antibody JNJ-61186372 enhanced killing of lung cancer cells
Source: MAbs. 2016 Oct 27;9(1):114–26. doi: 10.1080/19420862.2016.1249079 (PMC5240640; doi:10.1080/19420862.2016.1249079)

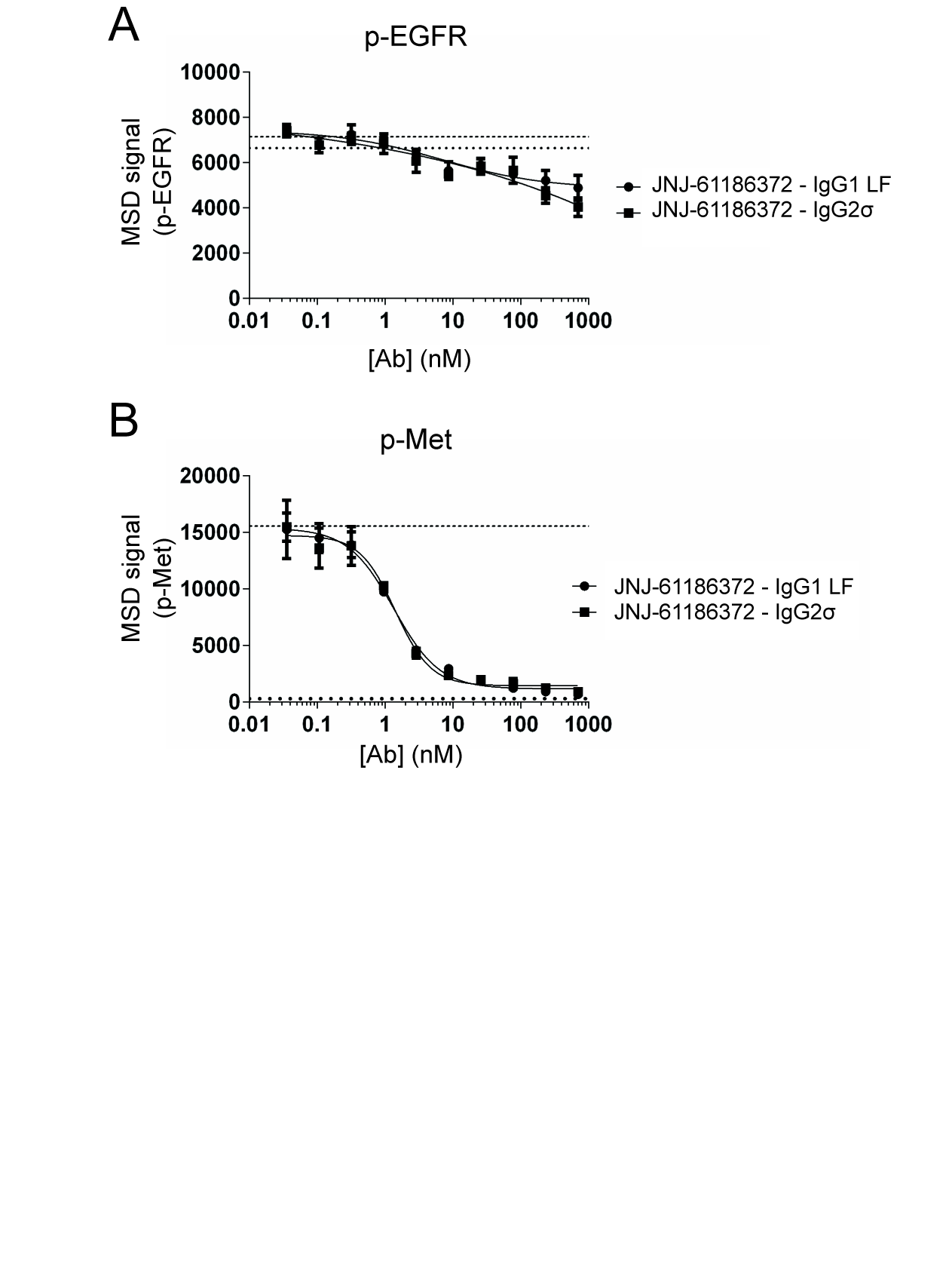

Supplement: Supplemental_Data.zip [file kmab-09-01-1249079-s001.zip › 2. Supplemental Figure 1.tif]
